# Supplementary material for: Impact of Salvage Surgery on Health-Related Quality of Life in Oral Squamous Cell Carcinoma: A Prospective Multi-Center Study
Source: J Clin Med. 2023 Oct 18;12(20):6602. doi: 10.3390/jcm12206602 (PMC10607572; doi:10.3390/jcm12206602)
Supplement: Supplementary file 1 [file jcm-12-06602-s001.zip › jcm-2607475-supplementary.pdf]

Table S1. Linear mixed model regression for global health status difference adjusted to baseline.

	Estimate	Lower CL	Upper CL	*p*-Value
Tumor stage: [1–2]	0	-	-	-
Tumor stage: [3–4]	12.403	−7.982	32.788	0.225
Gender: Man	0	-	-	
Gender: Woman	−6.486	−25.744	12.773	0.499
Age	0.959	0.072	1.846	0.035

Interpretation: For two patients of the same age and gender and with a tumor in stage 1–2 and 3–4, respectively, the self-reported global health status score is ca. 12.4 points higher for the patient with a tumor in stage 3–4 compared to the patient with a tumor in stage 1–2.

Table S2. Linear mixed model regression for pain in the mouth difference adjusted to baseline.

	Estimate	Lower CL	Upper CL	*p*-Value
Tumor stage: [1–2]	0	-	-	-
Tumor stage: [3–4]	−13.505	−33.728	6.717	0.184
Gender: Man	0	–	–	
Gender: Woman	6.877	−12.124	25.878	0.467
Age	−0.872	−1.725	−0.019	0.045

Interpretation: For two patients of the same age and gender and with a tumor in stage 1–2 and 3–4, respectively, the self-reported pain in the mouth score is ca. 13.5 points lower for the patient with a tumor in stage 3–4 compared to the patient with a tumor in stage 1–2.

Table S3. Linear mixed model regression for swallowing difference adjusted to baseline.

	Estimate	Lower CL	Upper CL	*p*-Value
Tumor stage: [1–2]	0	-	-	-
Tumor stage: [3–4]	−1.102	−19.758	17.553	0.905
Gender: Man	0	-	-	
Gender: Woman	−8.454	−25.893	8.984	0.332
Age	−0.315	−1.105	0.476	0.424

Interpretation: For two patients of the same age and gender and with a tumor in stage 1–2 and 3–4, respectively, the self-reported swallowing function score is ca. 1 points higher for the patient with a tumor in stage 3–4 compared to the patient with a tumor in stage 1–2.

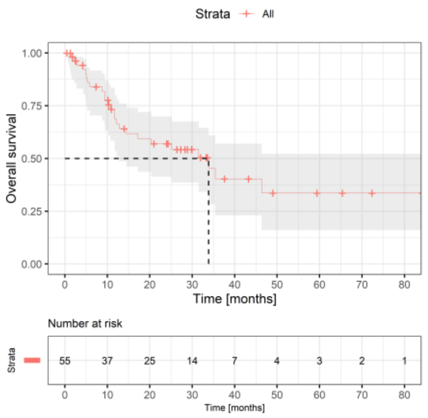

(**a**)

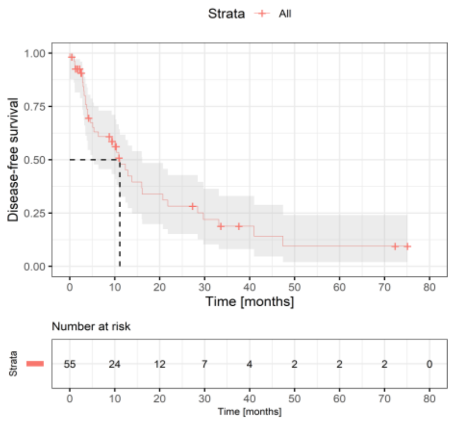

(**b**)

Figure S1 (**a**) Estimated overall survival of all patients, median overall survival 33.9 months. (**b**) Estimated disease-free survival of all patients, median disease-free survival 11.1 months
